# Extraction of Phenolic Compounds from *Populus* Salicaceae Bark

**DOI:** 10.3390/biom12040539

**Published:** 2022-04-02

**Authors:** Elsa Autor, Alfonso Cornejo, Fernando Bimbela, Maitane Maisterra, Luis M. Gandía, Víctor Martínez-Merino

**Affiliations:** 1 Department of Sciences, Universidad Pública de Navarra (UPNA), 31006 Pamplona, Spain; autor.105309@e.unavarra.es (E.A.); maitane.maisterra@unavarra.es (M.M.); lgandia@unavarra.es (L.M.G.); merino@unavarra.es (V.M.-M.); 2 Institute for Advanced Materials and Mathematics (InaMat2), Universidad Pública de Navarra (UPNA), 31006 Pamplona, Spain

**Keywords:** antioxidants, tannins, phenolics, Soxhlet, Folin-Ciocalteu, 2,2-Diphenyl-picrylhydrazyl, DPPH

## Abstract

Lignocellulosic residues have the potential for obtaining high value-added products that could be better valorized if biorefinery strategies are adopted. The debarking of short-rotation crops yields important amounts of residues that are currently underexploited as low-grade fuel and could be a renewable source of phenolic compounds and other important phytochemicals. The isolation of these compounds can be carried out by different methods, but for attaining an integral valorization of barks, a preliminary extraction step for phytochemicals should be included. Using optimized extraction methods based on Soxhlet extraction can be effective for the isolation of phenolic compounds with antioxidant properties. In this study, poplar bark (*Populus* Salicaceae) was used to obtain a series of extracts using five different solvents in a sequential extraction of 24 h each in a Soxhlet extractor. Selected solvents were put in contact with the bark sample raffinate following an increasing order of polarity: n-hexane, dichloromethane, ethyl acetate, methanol, and water. The oily residues of the extracts obtained after each extraction were further subjected to flash chromatography, and the fractions obtained were characterized by gas chromatography coupled with mass spectrometry (GC–MS). The total phenolic content (TPC) was determined using the Folin–Ciocalteu method, and the antioxidant activity (AOA) of the samples was evaluated in their reaction with the free radical 2,2-Diphenyl-picrylhydrazyl (DPPH method). Polar solvents allowed for higher individual extraction yields, with overall extraction yields at around 23% (dry, ash-free basis). Different compounds were identified, including hydrolyzable tannins, phenolic monomers such as catechol and vanillin, pentoses and hexoses, and other organic compounds such as long-chain alkanes, alcohols, and carboxylic acids, among others. An excellent correlation was found between TPC and antioxidant activity for the samples analyzed. The fractions obtained using methanol showed the highest phenolic content (608 μg of gallic acid equivalent (GAE)/mg) and the greatest antioxidant activity.

## 1. Introduction

For many centuries now, humanity has used the natural resources at their disposal and has tried to obtain valuable products from biomass, seeking to produce natural remedies to cure different illnesses and health-related problems, as well as to exploit plants and trees to obtain different commodities, energy, and all sorts of tools and manufactured items. The development of technologies for transforming biomass into valuable products dates back to 38,000 years ago [1]. The leaves and bark of the willow tree were used to treat pain as early as the fourth century BC, ultimately leading, in the 19th century, to the isolation of salicylates from different tree species and plants as active compounds to be used in the manufacture of commercial painkillers [2]. The industrial exploitation of rapid-growth and short-rotation crops of herbaceous species such as miscanthus [3], wheat [4], and camelina straw [5], and trees such as eucalyptus [5] and poplar [6], among others, has developed an important economic activity in the field of bioenergy [7] and in the development of biobased products in different sectors, namely the pulp and paper industry [8] and the manufacture of furniture and wood panels [6]. Such lignocellulosic materials are industrially exploited owing to their low cost, high productivity, and relatively easy production in considerable amounts in non-tropical weather [5].

The cultivation of these crops offers numerous advantages for developing the bioeconomy. Of particular interest is the development of the value chain of poplar (*Populus* sp.). According to Propopulus, a European association of growers, companies, and organizations that belongs to the poplar chain, poplar is a profitable species that can generate up to 800 €/Ha. [9]. It is a short-rotation species since the trees reach maturity in approximately 15 years, and this rapid growth makes their carbon fixation capacity greater than that of other species. With appropriate soil conditions, poplar trees can produce up to 20 tons of wood per hectare and year (on a dry basis) [7]. This type of agroforestry industry exploits the wood from the logs in the manufacture of boards, furniture, and other goods and wooden products including matches, chopsticks, and packaging for the agro-food industry, among others. Due to the optimal properties of poplar wood (lightness, color, lack of odor and taste, great homogeneity, and ease of processing), the main exploitation of poplar wood takes place in the plywood industry, being the main species used in the production of plywood in several European countries such as Spain, France, Italy, and Hungary [9].

In the industrial processing of poplar logs, one of the preliminary steps is debarking, in which great amounts of bark (9–10% of the tree mass [10]) are obtained as residue. Their current valorization is mainly oriented toward low value-added applications, such as low-grade fuel in pulp and paper mills [10]. To produce bioenergy out of these residues represents an inefficient way to valorize them because of the low value-added of the final use, in which the great chemical richness of poplar bark cannot be fully exploited. The development of biorefinery strategies for the full and optimal valorization of lignocellulosic residues such as poplar sawdust and bark is the best way to increase sustainability in the production of bio-based fuels and products, find renewable sources for aromatic compounds [11], and succeed in the development of a circular economy [4]. Recently, an alternative application of poplar bark was proposed as an efficient bio-based thermal insulation material in the field of “green building” [12]. The thermal insulation properties of bark were enhanced by means of an alkaline extraction treatment followed by lyophilization.

High value-added products can be obtained from poplar bark owing to its richness in phenolic compounds. Three main types of molecules can be obtained from barks: tannins, lignin, and cellulose [13], though bark generally presents a lower cellulose content in comparison to log wood [10]. In addition, valuable secondary metabolites can be found in poplar bark, such as nitrogenated and sulfated compounds, phenolics, and terpenoids [14]. In fact, poplar bark has been regarded as an important renewable source for bioactive compounds with great potential for valorization and capitalization [15]. Phenolic compounds possess very interesting characteristics from the point of view of their further use as additives, owing to their antioxidant properties [16].

The scientific literature proposes different alternatives for the valorization of bark residues from different tree species toward the production of higher value-added products, such as chemical compounds and materials. In fact, review articles can be found on this topic [15,17,18]. Most of the processing strategies for obtaining chemical compounds from barks can be classified into two main groups: (a) thermochemical methods, which imply the use of high temperatures (250–600 °C), such as solvolytic liquefaction [19] and pyrolysis [20], and (b) physicochemical extraction methods, typically carried out at much lower temperatures (80–150 °C) and making use of different solvents and chemical reagents. The latter should always be considered in the integral valorization of barks, thereby recovering the abundant amount of extractives present in the bark prior to their further processing by other methods [17]. Crudes obtained by solvolytic liquefaction or by pyrolysis (bio-oils) possess a high antioxidant capacity and have been proposed as additives to enhance biodiesel stability against oxidation [19,21].

Very few studies have been devoted to the extraction of phytochemicals from poplar bark [10]. Some of these studies focused on the use of different physicochemical extraction techniques for, firstly, conducting a characterization of the complex composition of poplar bark [22], and secondly, isolating certain compounds to further use them in different products and applications. As concerns characterization, studies that are oriented to extract and identify compounds from poplar bark generally use chromatographic methods and advanced separation techniques, such as high-performance liquid chromatography (HPLC) combined with analytical techniques such as nuclear magnetic resonance (NMR), mass spectrometry (MS) and infrared (IR) spectroscopy, among others.

It must be highlighted that isolating, purifying, and identifying bioactive molecules present in poplar bark is a challenging task since these molecules often represent less than 1% of the crude extract obtained [10]. In general, bark extracts obtained by acid hydrolysis present high glucose contents (60–70%), mainly derived from cellulose, as well as various sugars which constitute hemicelluloses: xylose (5–15%), arabinose (5–10%), galactose (3–4%) and mannose (3–4%). In some of the studies, it was found that certain flavonoids extracted from poplar bark, more precisely kaempferol, have high anti-inflammatory activity, which could explain the widespread use of poplar bark in the frame of ethno-medicine [22]. Also remarkable is the high content of polyphenols, with total contents that can range between 96 and 335 mg of gallic acid equivalents (GAE) per g of bark (on a dry basis) [15,23].

Only a few studies have been centered on the isolation and purification of extractives in poplar bark for their further use in different applications. In some cases, extractions were carried out by means of simple acid or alkaline hydrolysis. Viëtor et al. [24] adopted a sequential extraction method using different acid and alkaline aqueous solutions: boiling acidulated water (3 × 30 min, pH 5.5), 1% EDTA (2 × 6 h at 60 °C, pH 5.5), 1 M of NaOH/26 mM of NaBH_4_ (8 h at room temperature), and 4 M of NaOH/26 mM of NaBH_4_ (8 h at room temperature). The adopted sequential extraction yielded great amounts of pectic sugars, galactose, rhamnose, and arabinose, as well as xylans and xyloglucans. 

Furthermore, it has also been proposed to extract organic compounds from dry vegetal tissues by means of continuous extraction using Soxhlet extraction, using different solvents and taking into account the different affinities with the substances of interest. To that end, two studies can be highlighted as concerns the use of poplar bark as raw material. Devappa et al. [25] treated poplar bark in an industrial pilot plant using two distinct approaches: solvent extraction and autohydrolysis by steam explosion. Within the first route, different methods were explored which included extraction with organic solvents (ethanol, methanol, chloroform, diethyl ether, and acetone) and with water in a Soxhlet extractor, as well as different aqueous extractions at room temperature, including maceration, mechanical extraction by pressing the soaked bark residue obtained after the maceration, and subsequent treatment by steam explosion to obtain further extracts from the autohydrolysis of the matter. The Soxhlet extracts mainly contained flavonoids and derivatives of benzoic acid, including sakuranin and eupatoretin (extracted with acetone), ß-sitosterol (with diethyl ether), stigmast-4-en-3-one (with dichloromethane), and benzoic acid (with methanol). Aqueous extracts contained the following phytochemicals: benzoic acid, 2-methyl-cyclohexanone, palmitic, linoleic, and stearic acids, and sakuranin. 

Recently, Bremer et al. have studied the potential of poplar bark extracts as bio-based fungicidal additives [26]. Triterpenes, fatty acids, aldehydes, and alcohols were primarily the active fungicides in the Soxhlet extracts, the content of which was much dependent on the type of poplar species and on the age of the samples used. The presence of oligomeric sugars was also found in bark extracts. To that end, the authors concluded that a pre-extraction with water could be necessary.

In this study, a sequential extraction using five different solvents in a Soxhlet extractor was carried out using poplar bark derived from an agroforestry industry. The solvents were consecutively put in contact with bark samples for 24 h, in increasing polarity order (n-hexane, dichloromethane, ethyl acetate, methanol, and water), which was adopted to obtain extracts containing valuable compounds. The extracts were further fractionated by flash column chromatography, the fractions obtained were characterized by gas chromatography coupled to a mass detector (GC–MS), and their total phenolic content (TPC) and antioxidant activity were measured. 

## 2. Materials and Methods

### 2.1. Chemicals and Reagents

All high-purity liquid and solid chemicals were purchased from Carlo Erba (available at www.carloerbareagents.com, accessed on 29 March 2022), Fisher Scientific (available at www.fishersci.es, accessed on 29 March 2022), and Sigma-Aldrich (available at www.sigmaaldrich.com, accessed on 29 March 2022) and used as received. Thin-layer chromatography was carried out using Silica Pre-coated TLC sheets (Alugram Xtra SIL, available at www.mn-net.com, accessed on 29 March 2022) and revealed at 254 nm using anisaldehyde. High-purity gases were supplied by Nippon Gases, Spain (available at www.nippongases.com/es-es/, accessed on 29 March 2022).

### 2.2. Sample Preparation

Poplar bark (*Populus* Salicaceae) samples were provided by Garnica Plywood Inc. (Baños de Río Tobía, La Rioja, Spain) as a residue from the debarking process of poplar. All samples were collected in 2019. Upon their reception, the moisture in the sample was high, so in order to improve the preservation of the sample, the bark was dried on a stove at 103 °C in perforated trays for 24 h. Then, the samples were milled using an electric mill (UFESA MC0470), sieved to have particle sizes below 0.2 mm, and stored at room temperature. The ash content in the starting material was determined according to UNE-EN-14775 [27]. An amount of 1 g of the dried sample was weighed, put in a porcelain crucible, and placed in a muffle furnace. Then, the sample was heated to 250 °C at 5 °C·min^−1^ and held for 30 min. Next, the temperature was raised to 550 °C at 10 °C·min^−1^ and held for 120 min. The sample was then allowed to cool down until room temperature was reached and then weighed. 

Humidity was determined according to UNE-32-102 [28]. For this, ca. 1 g of the sample was placed in a preheated oven at 105 °C and was allowed to dry at this temperature for 60 min. After the time elapsed, the sample was removed, rapidly cooled down, and placed in a desiccator for 15 min. Once at room temperature, the weight was recorded. Both the ash and the humidity determinations were obtained by applying the weight loss formula.

### 2.3. Soxhlet and Chromatography Fractionation

20 g of milled poplar bark sample (particle diameter < 0.2 mm) was placed in a cellulose cartridge (30 × 100 mm and 8–15 µm nominal retention; Scharlab, Part number: CT32530100) and placed in the Soxhlet extraction system. The sample was successively extracted with 250 mL of n-hexane, dichloromethane, ethyl acetate, methanol, and water for 24 h with each solvent. Each of the extracts was concentrated by rotary distillation at 40 °C to yield an oily residue. 

Oily residues from each of the solvents were gathered and subsequently fractionated by flash chromatography using SiO_2_ (Silica Gel 60, 0.04–0.06 mm, 230–400 mesh, Scharlab) as a stationary phase and with an increasing polarity gradient of eluents depending on the extraction solvent polarity (see SI.1, Table S1). TLC for the different fractions obtained by flash chromatography was made using the same eluent in which the fraction was eluted (see SI) on Silica Gel Plates on aluminum foil (Alugram Sil G UV254, Macheray Nagel) and revealed using anisaldehyde and UV light (254 nm).

### 2.4. GC–MS Analysis

The qualitative analysis of selected extract samples was carried out in order to identify the compounds present in the oily residues after fractionation using flash chromatography (see Section 2.3). Analyses were performed in an Agilent 7890B gas chromatograph equipped with a 5977B mass spectrometry detector. Samples were injected in “splitless” mode with a PAL RSI 120 automatic injector. An HP-5MS capillary column [(5-phenyl)-methylpolysiloxane, 60 m × 0.32 mm] was used at the stationary phase. This stationary phase is commonly used in the analysis of phenolic compounds [11]. Helium was used as a carrier gas (104 mL·min^−1^) and the inlet injector was kept at 230 °C. The temperature program started at 50 °C and heating at 10 °C/min was carried out until a temperature of 325 °C was attained and then held for 15 min. Mass/charge (m/z) signals were obtained in full scan mode in the 10–500 a.m.u. range. The identification of the components in the extracts was assigned by the comparison of their retention times and mass spectra fragmentation pattern with NIST library v2.2. The semi-quantitative analysis of phenolic compounds and saccharides was performed using the relative percentage areas expressed as the integration of the compound signal referred to the whole integration of identified peaks at retention times (r.t.) between 7.0 and 26.0 min. The choice for this retention time interval was motivated by the solvent delay of the analysis method and the detection of a huge amount of compounds from column bleeding at retention times longer than 26 min.

### 2.5. Phenolic Content Determination

The total phenolic content (TPC) of the samples was determined by using the Folin–Ciocalteu method [29]. The calibration curve was prepared using gallic acid as standard. Briefly, from an aqueous or acetone:methanol 1:1 100 mg·L^−1^ gallic acid solution, a series of dilutions were prepared with high-purity deionized water or acetone:methanol 1:1 to obtain standard concentrations of 20, 10, 5, 2.5, and 0 mg·L^−1^. Amounts of 0.500 mL of the standard solution described above were placed in different vials protected from light. Next, 0.750 mL of 1 N Folin–Ciocalteu reagent was added to each vial, shaken, and then 0.750 mL of a 7.5% *w*/*v* sodium carbonate Na_2_CO_3_ solution was added. The resulting mixture was allowed to settle in dark for 2h. The absorbance of the standards was measured at λ = 765 nm in a Selecta V-110D spectrophotometer. A light white precipitate was observed in standards prepared using acetone:methanol 1:1 gallic acid solutions, which was removed using a 0.45 μm SPE filter prior to absorbance measurement.

Regarding the analysis of the different samples containing the extracts obtained from poplar bark, the procedure was similar: 2–3 mg of sample was diluted in 50 mL of high-purity deionized water in the case of ethyl acetate, methanol, and water extracts or in an acetone:methanol 1:1 mixture in the case of dichloromethane extracts. An amount of 0.500 mL of the sample solution was then mixed with the Folin–Ciocalteu reagent (0.750 mL, 1 N) and a solution of sodium carbonate (7.5% *w*/*v*, 0.750 mL). The resulting mixture was allowed to settle for 2 h in the dark, and the absorbance was measured at λ = 765 nm. In the case of dichloromethane extracts dissolved in acetone:methanol 1:1, a light white precipitate appeared, which was removed using a 0.45 μm SPE filter.

### 2.6. Determination of the Antioxidant Activity

The antioxidant activity of phenolic compounds was measured by the 2,2-Diphenyl-1-picrylhydrazyl (DPPH) method as described elsewhere [30,31]. The calibration curve was prepared by using ascorbic acid as the calibration standard. Briefly, 0.5 mL of ascorbic acid standard at 40, 20, 10, 5, 2.5, and 1.25 mg/L was mixed thoroughly with 1.5 mL of DPPH–methanol reagent (4.5 mg of DPPH in 50 mL of methanol) and allowed to stay at room temperature in the dark for 5 min prior to measuring the solution absorbance at λ = 517 nm in a Selecta V-110D spectrometer.

Once the calibration curve was established, 0.500 mL of diluted extract (60 mg/L) was mixed with 1.500 mL of the DPPH–methanol reagent. The absorbance of the sample at λ = 517 nm was measured every 5 min for a total reaction time of 70 min.

## 3. Results and Discussion

Amidst all the poplar varieties, *Populus* Salicaceae is used in the production of plywood in the most important factory in the region of La Rioja in northern Spain. *Populus* Salicaceae is debarked upon receipt and the bark is used as a low-grade fuel. The extraction and fractionation of phenolic compounds can be of great interest for the valorization of this industrial residue.

Bark samples are extremely wet when received, which could cause mold growth. It is well known that drying inhibits the enzymatic degradation of plant biomass and limits microbial growth. Drying conditions, whatever they are, will affect the sample to some extent. Ambient air-drying has traditionally been used to preserve medicinal herbs because low temperatures have been considered to inhibit the degradation of the active ingredients. However, this is a slow method, and the continuation of metabolic processes at the beginning of drying may result in losses in material properties. Thus, in a recent study, the effects of thermal drying on the contents of condensed tannins and stilbenes in Norway spruce bark were analyzed. Drying at 70 °C for 24 h reduced moisture from 65% to ca. 25%, whereas the condensed tannins content was reduced from ca. 35 mg·g^−1^ to 23 mg·g^−1^ [32]. A similar study on willow bark concluded that drying at 70 °C in a tray did not cause significant changes neither in the phenol nor in the flavonoids or the tannin contents [33]. Drying temperatures as high as 120 °C have been tested in the extraction of phenolic compounds from lime waste, which affected the extraction patterns of phenolic compounds [34]. Bark samples were dried at 103 °C for 24 h in perforated trays to 7% moisture content. Certainly, drying at high temperatures can oxidize polyphenols, causing the loss of some of their antioxidant properties. In this case, the degradation of certain labile components of the biomass may have occurred. For instance, furfural or 5-hydroxymethylfurfural, which are typically produced upon saccharide cyclodehydration at high temperatures, were detected in some of the extracts (*vide infra*).

After drying, the moisture content of poplar bark was 7.0 ± 0.5%, which is slightly lower than that reported in previous studies by Scott et al., who reported moisture contents of around 7.55% [35]. The ash content was 5.8 ± 0.5%, much higher than that reported in the literature for poplar wood, which is usually in the 1.0–4.5% range [19,35,36,37], but in the 5–10% range for different species of poplar bark, as reported in the literature [38].

This study was focused on the detection of phenolic compounds in *Populus* Salicaceae bark extracts and the determination of their total phenolic content (TPC) and antioxidant activity (AOA). A wide variety of chemical compounds can be extracted from poplar bark, therefore, a previous fractionation was performed. One of the most straightforward methodologies to isolate compounds from bark consists of Soxhlet extraction. This methodology presents many advantages. First, it is a straightforward methodology, which does not need any special equipment. Second, it is highly reproducible. Third, successive extraction runs can be performed using different polarity solvents and in different polarity sequences. Last, but not least, the extract can be easily obtained upon solvent distillation, allowing solvent recovery, which increases both the economic and environmental sustainability of the overall process.

In a previous work (not published) carried out in our research group, the extraction of antioxidants from pine bark using Soxhlet extraction was studied [39]. In that case, it could be observed how the nature of the solvent and the order of the extraction solvents could influence the extraction yields. Four different solvents, n-hexane, dichloromethane, ethyl acetate, and methanol were used in successive extractions of active compounds from pine bark. The best results, in terms of extracted mass yields, were achieved when these solvents were used in increasing order of dipole moment for hexane, dichloromethane and ethyl acetate, and finally methanol, due to its ability to form hydrogen bonds. Consequently, when the four successive extractions were carried out for 24 h, the overall mass yield was 74.1%, methanol being the solvent that provided the highest extraction yield (64.9%). Unfortunately, when methanol extracts were analyzed by GC–MS, saccharides were the most abundant compounds by far, and only traces of 2,4-Di-*tert-*butylphenol were detected.

**Scheme 1:**
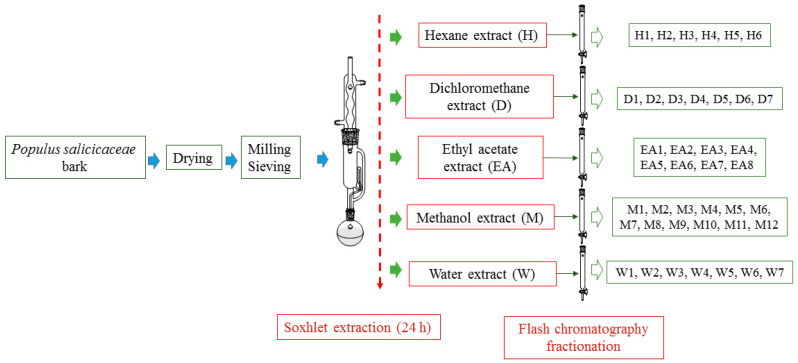
Sample preparation procedure.

In the present study, the same increasing solvent polarity was maintained in Soxhlet extraction. In order to optimize the extraction of the target compounds, the closest contact between the sample and the extract must be ensured to provide higher extraction yields. Owing to this, a relatively small particle size was chosen, dp < 0.2 mm, for the extraction experiment. Milled bark samples were successively Soxhlet extracted with n-hexane, dichloromethane, ethyl acetate, and methanol in 24 h rounds. It can be expected that hexane may extract most hydrophobic compounds, mainly lipids, waxes, or terpenes that can be found in poplar bark [40]. Middle-range polarity solvents, such as dichloromethane and ethyl acetate were included in the extraction sequence with the aim of obtaining more specific fractionation to extract, at least, part of the phenolic compounds. The resulting extracts from each of the solvents were further fractionated by flash chromatography using increasing polarity eluents (see SI.3, Table S2 for n-hexane extracts, Table S3 for dichloromethane extracts, Table S4 for ethyl acetate extracts, Table S5 for methanol extracts and Table S6 for water extracts, and Scheme 1).

Because of environmental concerns, water is the preferred solvent for the extraction of polar compounds. Nevertheless, extraction with water leads to low pH values that cause the formation of non-soluble precipitates due to the auto-condensation reactions of the extracted tannins [41], as well as the formation of covalent bonds with the cellulosic matrix [17], which limits the extraction yield. Although 70% ethanol is commonly used in batch extraction processes, methanol was used in the Soxhlet extraction process. Methanol, although being toxic, presents higher polarity than ethanol, which may increase the extraction yields, and its boiling point is noticeably lower (65 °C vs. 78 °C). Methanol is used in many reported processes for extraction from bark [3]. Finally, water was added at the end of the extraction process to ensure the complete extraction of polar compounds. Indeed, phenolic compounds with antioxidant properties must be present in bark as hydrolyzable tannins [42]. They can be released by extraction using methanol, given their high solubility in this polar protic solvent [43]. Apart from dipole moment and dielectric constant, it is important to notice that all the selected solvents presented low or relatively low boiling points (40 °C to 80 °C). Additionally, the boiling points for hexanes, ethyl acetate, and methanol are rather similar (69 °C, 78 °C, and 65 °C).

Table 1 shows the individual extraction yields using the different solvents, as well as the total phenolic content (expressed as μg of gallic acid equivalents (GAE)/mg) and the antioxidant activity (expressed as the μg of ascorbic acid equivalents (AAE)/mg). Overall extraction yields from poplar bark ranged from 20% to 25% (on a dry mass basis) in all rounds. Extraction yield values were highly reproducible in five replicated Soxhlet extractions (24.3 ± 2.0%). However, this yield is noticeably lower than that measured in our previous study with pine bark under the same conditions (74%), which can be explained by the presence of highly methanol-soluble resins in pine bark [22]. Extraction yields higher than 44% have been reported in the extraction of chestnut bark, though using subcritical water [44].

As expected, the extraction yields for each of the solvents were different. Thus, hexane and dichloromethane extracted only 2.1% and 1.1% of the bark mass. Similar extraction yields were obtained when ethyl acetate (2.8%) and water (2.6%) were used as extraction media. As expected, the highest extraction yields were obtained when methanol was used as the extraction solvent, reaching 15.6% (dry basis), which is in agreement with previous studies on pine bark extraction [12], which suggests that most of the polar compounds were extracted with methanol. Indeed, it can be expected that most phenolic compounds with antioxidant properties contained in bark are polar and therefore highly soluble in methanol. The extraction yields obtained with the different solvents suggest that most of the extractable compounds are soluble in methanol. For some of them, such as, for instance, gallic acid or salicylic acid, derivatives may be present in poplar bark [42] as hydrolyzable tannins; hence, they can be released either by methanol or by pressurized hot water [45], though the latter would require more severe conditions. Indeed, it has been reported that 100% of the tannins contained in pine bark can be extracted using methanol [43].

The Total Phenolic Content (TPC, Table 1) for crude solvent extracts was measured using the Folin–Ciocalteu method [46,47]. Ethyl acetate (EA) and water (W) extracts presented moderate TPC values, ca. 120 μg GAE/mg, while the TPC for methanol extracts (M) was slightly lower, 84 μg GAE/mg, due to the presence of saccharides (*vide infra)*. Such values are lower than the TPC values of the aerial part of *Salvia bicolor* [48]. The TPC values for hexane (H) and dichloromethane (D) extracts were surprisingly high, 343 μg GAE/mg and 172 μg GAE/mg, respectively. However, it must be noted that these samples were completely insoluble in water and the measurement was made in an acetone:methanol 1:1 mixture, which did not provide comparable results with those of EA, M, and W.

To evaluate the antioxidant activity (AOA) of the extracts, first, the decay of the absorbance of DPPH· was evaluated in the absence of any extract and/or ascorbic acid (see SI.2, Figure S1). It could be observed in blank samples how from minute 40 onward, the absorbance at 517 nm decayed. This decay in the absorbance is due to the reaction of the DPPH radical with the proton of the solvent; as can be expected, the decay in absorbance is faster in water than in methanol:acetone because of its higher donor character. Therefore, the antioxidant capacity expressed in terms of the activity of ascorbic acid was calculated as a function of the maximum absorbance decay at 517 nm during the first 30 min.

As shown in Table 1, the AOAs of the extracts obtained with dichloromethane, ethyl acetate, and methanol were very similar, around 310 μg of AAE/mg, followed by those extracts obtained using water, 193 μg of AAE/mg. These AOA values were higher than those obtained in ethanol and ethanol:water extracts from pine bark [31]. D extracts presented high antioxidant activity (312 μg AAE/mg) but, as in the case of TPC determination, the sample was not soluble in water and, therefore, the results are not fully comparable. The lowest AOA was obtained for those extracts obtained with hexane, 122 μg of AAE/mg. Although it could be expected that most phenols and polyphenols were extracted in methanol, in our previous work on pine bark, it could be observed that almost no phenolic compounds were detected in this fraction, and most of the detected compounds corresponded to saccharides [39].

**Figure 1:**
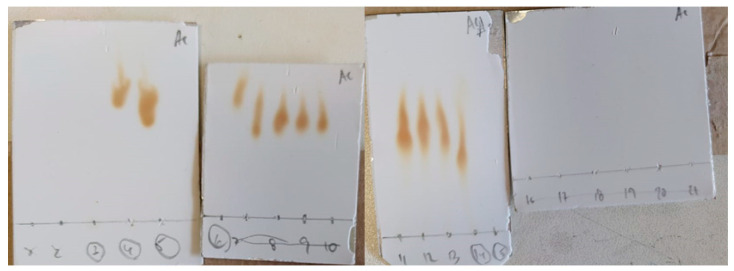
TLC plates used in the fractionation of ethyl acetate extract.

Overall, the TPCs and AOAs obtained in this work using poplar bark can be regarded as promising. However, in the case of methanol extracts, it can be considered that the fractionation of the extracts may lead to much higher TPC and AAO, as most saccharides are removed. Extracts from different extraction solvents were gathered and then fractionated according to their polarity by flash chromatography using eluents with increasing polarity gradients (see Scheme 1 and SI.3, Tables S2–S6). H extracts were eluted using hexane:ethyl acetate 1/2 and ethyl acetate; D extracts were eluted using dichloromethane:ethyl acetate in different ratios, from 7:3 to 1:5 and ethyl acetate. EA extracts were eluted using dichloromethane:methanol in different ratios, starting from 7:3 then 1:3, and finally methanol. Given the amount of mass of dry M extracts, these were eluted starting from dichloromethane:ethyl acetate, then dichloromethane ethanol, and finally methanol. Finally, W extracts were eluted using methanol and propan-2-ol. As a result, different fractions with homogeneous volumes arising from extracts’ fractionation were collected (34, 57, 32, 85, and 41 for H, D, EA, M, and W, respectively). Fractions were checked by TLC and gathered into homogeneous fractions (see SI, Tables S2–S6 and Figure 1).

Chromatography fractionation provided only 19% of the original D extract, whereas 64% and 48% of the extract mass was recovered for EA and M after column chromatography. Column chromatography fractionation with the increasing polarity gradient described above proved to be successful. Aqueous extract fractions, W, did not present any phenolic content. Methanol extracts presented good-to-excellent phenolic contents ranging from 67 μg GAE/mg to 608 μg GAE/mg (Table 2) being, in most of the fractions, noticeably higher than in the crude methanol extract. Ethyl acetate fractions EA1, EA2, EA3, and EA4 (234, 371, 245, and 200 μg GAE/mg, respectively) also presented higher TPCs than in the overall EA extract (124 μg GAE/mg), but in the last eluted fractions EA5, EA6, EA7, and EA8 (12, 83, 82, and 45 μg GAE/mg), the TPCs were lower. A similar trend can be observed in methanol extracts fractionation, where the TPC was noticeably higher in fractions M1 and M3–M7 (608, 225, 331, 242, 240, 286 μg GAE/mg) than in the overall M extract and fractions M8, M9, M10, and M11 (102, 105, 98, and 67 μg GAE/mg). The TPC value for M2 is somewhat low (2 μg GAE/mg), but it has to be noted that the mass for this fraction was only 7 mg, which is due to it being the end of one phenolic fraction. This suggests that most of the phenolic compounds are concentrated in the first eluted chromatography fractions, whereas more polar fractions (E5–E8, M8–M11) are mainly constituted of non-active compounds. Dichloromethane fractions presented similar TPCs in the fractionated extracts (149–240 μg GAE/mg) than in the crude extract D (172 μg GAE/mg). This suggests that in dichloromethane extracts, no saccharides were extracted, thus chromatography fractionation would not be necessary in this case. As concerns the antioxidant activity, D2 presented lower AOA values than the whole extract (10 μg AAE/mg and 308 μg AAE/mg), while the highest measurable antioxidant activity was found for D4 (240 μg AAE/mg). Despite these good values for dichloromethane extract and its corresponding fractions, it is worth noting that the overall extraction yield was just 1.1% and that, after fractionation, only 19% of the crude extract mass was recovered (0.2% overall yield).

Regarding ethyl acetate fractionated samples, the AOA increases in EA1–EA4 (499, 617, 503, and 468 μg AAE/mg) compared to crude EA and EA5–EA7 (324, 74, 300, and 337 μg AAE/mg), where the TPC also decreased. In the case of methanol fractionation, the AOAs for M1 and M3–M10 (634, 713, 793, 678, 568, 485, 342, 358, and 356 μg AAE/mg) were noticeably higher than for the original M extract (261 μg AAE/mg). Surprisingly, high antioxidant activities were found for M9 and M10 (358 and 356 μg AAE/mg). Interestingly, the phenolic content in the methanol extracts decreased steadily from those eluted with less-polar eluents to those eluted with more-polar eluents; thus, the highest phenolic contents were found for the first eluted fraction, 608 μg GAE/mg, and then decreased to 242 μg GAE/mg in M5 and 67 μg GAE/mg in M11. The TPC content in ethyl acetate fractions was, however, more constant, ranging from 240 μg GAE/mg in EA1 and EA3 to 370 μg GAE/mg in EA2, which indicates that most of these hydrolyzable tannins may have been eluted in the first chromatography fractions using mixtures of dichloromethane/ethyl acetate and dichloromethane/methanol.

Despite being the sample with the highest phenolic content, M1 did not show the highest antioxidant activity; 634 μg AAE/mg against 793 μg AAE/mg for M4 (331 μg GAE/mg). These results could be attributed to the presence of other families of compounds with antioxidant properties, such as flavonoids [49]. Excluding M2 for the reasons explained above, the AOA increased from M1 to M4 and then steadily decreased to 356 μg AAE/mg in M9 and M10, following the same evolution as the phenolic content.

The AOA in EA fractions, however, seemed to be more related to the phenolic content (Table 2), reaching its highest activity in EA2 (617 μg AAE/mg), which also showed the highest content in phenolic compounds (371 μg GAE/mg), followed by EA3 (503 μg AAE/mg) and EA1 (499 μg AAE/mg). Subsequent fractions from ethyl acetate extracts presented a steady decrease in antioxidant activity, 468 μg AAE/mg in EA4 and 74 μg AAE/mg in EA5, which was at the end of the most important phenolic fraction.

**Figure 2:**
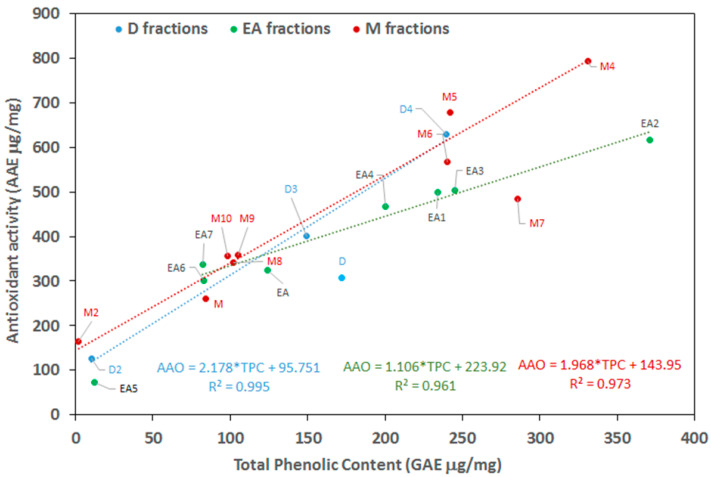
Plot of total phenolic content (TPC) and antioxidant activity for the different fractions after the chromatography of the crude extracts. H, D, EA, and M account for hexane, dichloromethane, and methanol, respectively. Figures account for elution order upon chromatography fractionation, with 1 being the first eluted fraction. Values for D, EA5, M1, and M7 are excluded from the calculation.

AOA and TPC are represented in Figure 2. It can be observed that nice correlations between AOA and TPC are observed for the fractions from the different extracts when the values for D, EA5, M1, and M7 are excluded. The effect of fractionation is clearly shown for the EA and M extracts, showing strong differences between the more polar eluted fractions that concentrate most of the phenolic compounds and the less polar fractions.

TPC and antioxidant activity are strongly dependent both on the raw material and the extraction solvent. The as-measured TPC and antioxidant values were noticeably higher than those obtained after extraction with ethanol from pine bark [31] in D, EA, and M. This difference is even more significant upon chromatography fractionation, as in the case of EA2 and EA3 and M3, M4, and M5. TPC was also in the range of that obtained using more sophisticated methodologies, such as the thermovacuum pretreatment of poplar bark [23]. The TPC values are also higher than those found for beech or spruce bark extracts obtained with ultrasound-assisted extraction [50] and in the same range as those found for the hydroalcoholic extracts of the bark of *Albizia niopoides* trees [51].

The TPC and AOA were surprisingly high for samples M8–M11, where, according to GC–MS analysis, phenolic contents are low (Table 3), while saccharides were detected in high amounts by GC–MS. Aside from the possibility of the presence of polyphenols and flavonoids that are not detected by our analysis method, it is also known that reducing sugars may cause interference in the Folin–Ciocalteu method [52,53,54]. In addition, it has also been reported that Maillard reaction compounds derived from that may cause TPC and AOA values to be much higher than expected [55]. This high content of saccharides and furfural and derived compounds may also be the origin of the lower TPC and AOA of EA and M extracts compared to those found for dichloromethane fractions that did not present saccharides in the GC–MS analysis.

**Figure 3:**
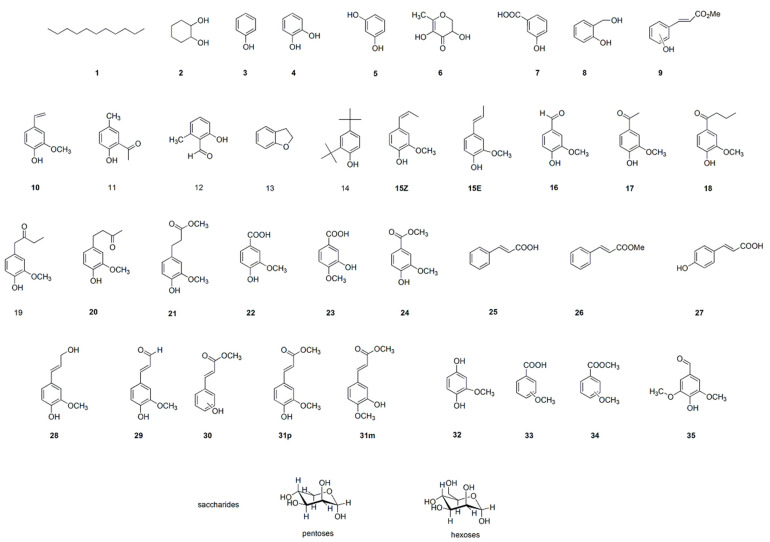
Chemical formulas of the compounds identified by GC–MS analysis. Numbering corresponds to peak numbers from Table 1.

Selected extracts were qualitatively analyzed by GC–MS (Table 3). The chemical formulas of the compounds identified are presented in Figure 3. Given that the highest extraction yields were obtained when using ethyl acetate and, especially, methanol, this analysis was focused on samples derived from the corresponding fractions of the extracts obtained with these two solvents. Nonetheless, samples from a water fraction (W3) and a hexane fraction (H4) were also analyzed by GC–MS. However, no chromatographic peaks could be detected with these two samples under our analysis conditions. The as-reported relative percentage areas are expressed as the integration of the compound signal referred to as the whole integration of identified peaks at retention times (r.t.) between 7.0 and 26.0 min. The choice for this retention time interval is motivated by the solvent delay of the analysis method and the appearance of compounds from column bleeding at retention times longer than 26 min. Similarly, the cumulative percent areas for saccharides for methanol extracts are presented in Table 3. GC–MS is not suitable for the determination of saccharides that must decompose, at least partially, in the GC injector. Pentoses and hexoses are identified as mannose or arabinose by the NIST 2.2 library. Therefore, this family of compounds is presented as saccharides and the contents in the fractions are consistent enough (*vide infra)* to show their increasing amount in methanol fractions eluted with more polar eluents (M5–M10).

**Figure 4:**
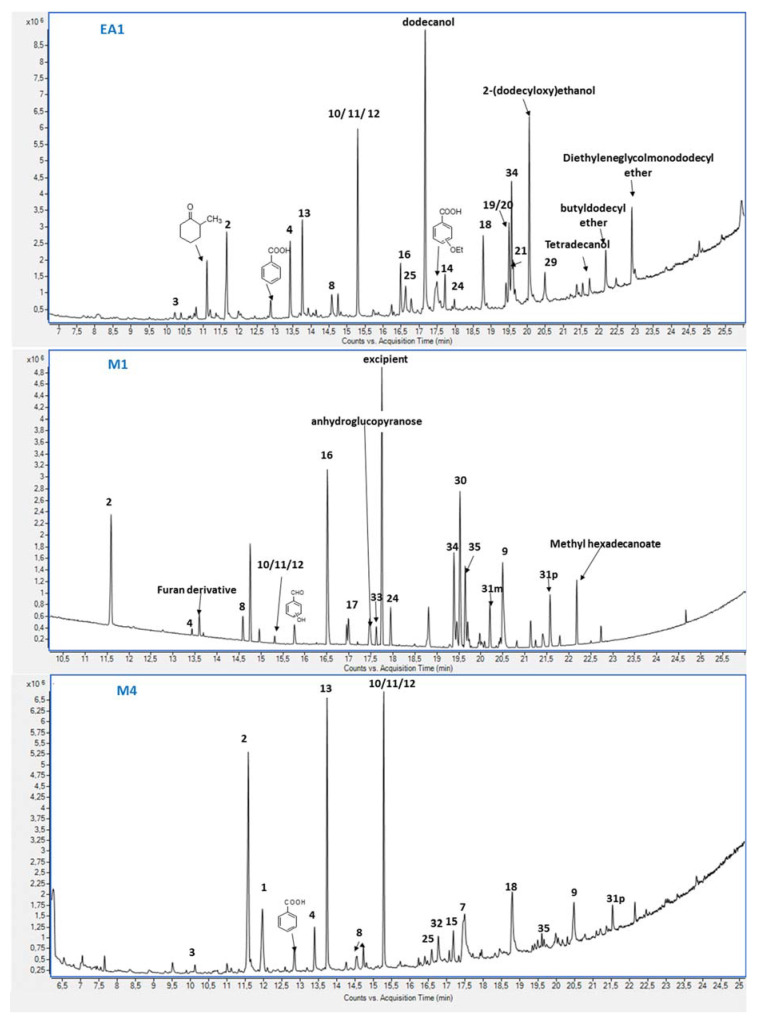
GC–MS chromatograms for samples EA1 (**Top**), M1 (**Middle**), and M4 (**Bottom**). Numbering corresponds to peak numbers from Table 1.

A semi-quantitative estimation of the substances, based on the comparison of peak areas, was performed. Only phenolic compounds described in the literature, as well as undecane, cyclohexan-1,2-diol, and saccharides were chosen for the discussion of the results. This does not disregard the presence of polyphenols, whose identification must be made after the derivatization of the sample or using liquid chromatography, but their identification is out of the scope of this study. As can be observed in Figure 4, the analysis conditions provided a nice separation of the aromatic compounds that allowed their identification using the NIST 2.2 library.

The effect of the solvent sequence in Soxhlet extraction and chromatography fraction was evidenced in the compounds that could be identified in the different GC–MS analyses (Figure 3, Figure 4, Table 2). Besides aromatic compounds, glycerol and glycerol derivatives such as glyceraldehyde dimer (r.t. 8.02 min) or glycolaldehyde dimethyl acetal (r.t. 7.65 min) were detected in the EA fractions. These compounds may come from the hydrolysis or trans-esterification of lipids. Dihydroxyacetone (r.t. 8.86 min), a compound that is used in the cosmetics industry in the formulation of self-tanning products [56], was also detected. Glycerol was far more abundant in EA8, reaching up to 13%A, and even noticeable amounts of fatty acid esters such as methyl hexadecaonoate (r.t 21.56 min) or methyl 9-octadecenoate (rt. 23.89 min) could be identified, with relative values of 1.9%A and 5.0%A, respectively, in this fraction. Long-chain alcohols such as 1-dodecanol or derivatives such as 2-(dodecyloxy)ethanol, dodecanal (r.t. 20.06 min), or n-nonadecanol (r.t. 19.55 min), probably from wax transesterification, were also detected in most EA fractions. Increasing amounts of pentoses and hexoses, mainly identified as arabinose and mannose by the NIST 2.2 library, were found in those methanol fractions eluted with more polar eluents (M5, M6, M7, M8, M9, and M10) and are gathered as saccharides in Table 3. Furfural and 5-hydroxymethyl furfural, which are usually formed in acidic conditions from hexoses and pentoses, respectively, have already been found in methanol fractions.

Long alkyl chain compounds, namely undecane (peak #1) or undecane isomers, were found in the fractions eluted with low polarity solvents from ethyl acetate and methanol extracts, accounting for ca. 8%A in all the analyzed methanol fractions. Long-chain alcohols such as 1-dodecanol and long-chain carboxylic acids that may come from wax hydrolysis were also detected (11.4%A) in the first fraction from ethyl acetate, EA1. The detection of these compounds in EA extracts reveals that they were not completely removed in hexane and dichloromethane extractions. Similarly, cyclohexane-1,2-diol 2 was detected in ethyl acetate (5.0%A, 6.3%A, 9.1%A, and 2.5%A in EA1, EA2, EA3, and EA4) and in methanol extracts, ranging from 15.0% in the first eluted fractions to 0.8%A in M10. Despite cyclohexan-1,2-diol not being a phenolic compound, the extraction of this compound is also of great interest in the chemical industry because of its possible application as a solvent in the fabrication of resins, coloring agents, plasticizers, or fire-retardant agents [57]. 2,3-dihydrobenzofuran (peak #13) was also found in all EA analyzed fractions in significant amounts (2.7%A–10%A) and in all methanol extracts, except for M1. However, although #13 presented a noticeable signal in M4 (12.8%A), it was drastically reduced in the rest of the methanol fractions (*ca.* 2.0%A–4.0%A).

The GC–MS analysis of dichloromethane extracts was focused on sample D3, since this was the only fraction with a significant mass (55 mg), while other fractions presented very low masses (see SI.3). Aside from compound #13 (0.5%A) and cyclohexan-1,2-diol (peak #2, 7.7%A), several phenolic compounds could be detected in D3, which could explain its high TPC and AOA: resorcinol (peak #5, 0.8%A), salicylic acid and salicyl alcohol (peak #8, 3.2%A), vanillin (peak #16, 1.1%A), di-*tert-*butylphenol isomers (peak #14, 1.1%A), butyrovanillone isomers (peaks #18, 19 and 20, 2.7%A), and syringaldehyde (peak #35, 1.0%A). Interestingly, cinnamic acid (peak #25) was the main compound in the GC–MS profile for D3 (10.8%A). Long alkyl chain alcohols such as 1-dodecanol or tetradecanol, as well as methyl octadecenoate, were particularly relevant in this sample, which evidenced that waxes are also efficiently extracted with dichloromethane. In contrast, neither saccharides (1.0%A) nor furfural derivatives (1.8%A) were significant.

As expected, a wide variety of phenolic compounds could be found in ethyl acetate and methanol fractions. Up to 31 phenolic compounds were detected in ethyl acetate extracts and up to 26 in methanol extracts (Table 3). Using ethyl acetate as the solvent yielded a wider diversity in terms of phenolic compounds despite being the sample extracted from poplar bark with the lowest mass. Phenol (peak #3) could only be found as a trace in EA1 and M4 (0.5%A), whereas catechol (peak #4) could be found in EA1–EA4 (3.2%A, 1.7%A, 2.7%A, and 0.9%A) and in M4 and M5 (2.6%A and 7.3%A). Catechol has also been found in poplar sawdust hydrolysis and in lignin depolymerization processes [58,59], as well as in poplar bark extracts, together with salicylic alcohol [26]. Indeed, catechol and salicylic alcohol are considered the main components in poplar bark extracts [25]. Many applications have been found for catechol in phytochemistry and in the pharma industry [58], as well as in the fuel industry as an antioxidant additive in biofuels [16].

4-vinyl-2-methoxyphenol (peak #10) or the corresponding isomer, 1-(2-hydroxy-5-methylphenyl)ethanone (peak #11), could be detected in all EA fractions analyzed. The EA1 and EA3 fractions presented the highest relative contents (7.1%A and 8.6%A), whereas EA2, EA4, and EA8 samples had similar contents of these compounds (2.2%A, 2.3%A, and 2.7%A, respectively). Compounds #10 and #11 were also present in most of the methanol fractions (12.5%A, 8.8%A, 4.1%A, 4.6%A, and 2.0%A in M4, M5, M6, M7, and M10, respectively). Isomer #10 is a very interesting compound, as it has found wide application in the food and pharma industries because of its anti-inflammatory properties [60]. However, isomer #11 is more probable from a biological point of view. Other phenolics with known antioxidant properties such as di-*tert-*butylphenol (peak #14), were found in EA1, EA4, and EA8 (1.6%A, 4.0%A, and 13.9%A).

Vanillin (peak #16), a phenolic compound with wide applications as a flavoring agent in the food industry, has also been detected in other bark extracts [15,22]. Vanillin was detected in EA extracts (2.1%A, 1.5%A, and 0.6%A for EA1, EA2, and EA4), but only in one methanol sample (M1, 8.0%A). As in the case of the dichloromethane extract, butyrovanillone isomers #18, #19 and #20 were detected in EA1–EA4 (3.6%A, 2.3%A, 7.4%A, and 2.0%A, respectively). In methanol, vanillin was only detected in M1 (8.0%A), together with guaiacyl ketone (peak #17, 0.8% A). Compound #18 was also detected in M4 and M5 (6.2%A and 5%A, respectively), but methyl ester #21 was only found in EA1 (4.6%A). Vanillic acid (peak #22) was detected in EA1–EA3 (0.2%A, 0.6%A, and 1.4%A, respectively) but not in the methanol extracts, whereas its isomer #23 was detected in EA1 and M5 (0.1%A and 0.5%A). Finally, vanillic acid methyl ester (peak #24) was detected as a trace compound in EA1 and in noticeable amounts in M1 (1.5%A). Cinnamic acid (peak #25) was detected in EA1–EA4, ranging from 1.4%A to 5.3%A, and also in M5, M6, and M8, though in lower amounts (0.2%A–0.6%A). However, coumaric acid (peak #27) could only be detected in ethyl acetate fractions EA2 and EA3 (1.5%A and 2.1%A), whereas the corresponding methyl ester (peak #30) could be found in EA8 (2.9%A) and M1 (6.0%A). Conyferyl aldehyde (peak #29) could be found in EA1–EA3 in noticeable amounts (1.6%A, 1.5%A, and 1.2%A) whereas the corresponding alcohol (peak #28) was only detected in EA4 (1.0%A). None of them could be detected in methanol extracts. Ferulic acid methyl esters, Methyl 3–(4-hydroxy-3-methoxy)prop-2-en-oate (peak #31p), and its isomer (peak #31m) were detected mainly in EA8 and M1 (0.9%A, 4.1%A), which indicates that this compound is well-extracted in both solvents. Ferulic acids and derivatives have also been detected in poplar bud extracts [61].

Other compounds, such as ethoxybenzoic acid (peak #33), were found in EA1 and EA2 (0.6%A and 6.0%A). Syringaldehyde (peak #35) was also found in EA1–EA3 (*ca.* 0.7%A) and detected in M5 as a trace. Finally, at long r.t. (29.985 min), traces of a compound that was identified as sakuranin by the NIST library were detected. Although the assignation could not be completely confirmed, this compound cannot be disregarded, as it has been reported in previous studies on bark extracts, especially in methanol and acetone [22,25,62].

The relatively high amount of glycerol derivatives, long-chain carboxylic acid derivatives, and alcohols suggest that both waxes and lipids were extracted from the bark using ethyl acetate due to incomplete lixiviation during hexane and dichloromethane treatments. Indeed, long-chain acid derivatives such as 9,12-octadecadienoic acid (r.t. 24.17 min), methyl 9-octadecenoate (r.t 23.89 min), hexadecanoic acid (r.t. 22.50 min), palmitoleic acid (18.90 min), as well as long-chain aliphatic alcohols were detected in dichloromethane extracts. Glycerol and glycerol derivatives could also be detected, although in noticeably lower amounts in methanol extracts.

Similarly, the presence of long-chain alkanes in ethyl acetate and methanol can be surprising at first sight. However, such alkanes have already been found in essential oils from plants obtained by hydrodistillation [63], as well as in poplar bud extracts [64]. Thus, these alkanes may not be completely extracted in the most hydrophobic fractions. Similar reasoning can be applied to long-chain alcohol and long-chain carboxylic acid derivatives in EA fractions that, however, come from waxes that are not totally removed during hexane and dichloromethane extraction.

Cyclohexane-1,2-diol (peak#2) and 2,3-dihydrobenzofuran (peak #13) could be detected in most of the ethyl acetate and methanol fractions. The presence of such cyclohexane-1,2-diol was somewhat expected, given that it is characteristic of some arborous species to have an accumulation of cyclohexane-1,2-diol glycosides and even glucosynolates [65], which may be cleaved upon the fractionation process or even during the drying step. The mass spectra for compound #13 also matched that of 4-methylbenzaldehyde. Although other aldehydes such as benzaldehyde and salicylaldehyde have already been detected in hexane extracts from poplar root, 4-methylbenzaldehyde could be synthesized from preformed salicinoids, a class of Salicaceae-specific phenolic glycosides derived from salicyl alcohol [23,66]. However, from a biochemical point of view, it is much more reasonable that compound #13 corresponds to 2,3-dihydrobenzofuran, given that benzofuranic compounds are derived from flavonoids [26,67].

Table 4 compiles the cumulative %A for the different families of aromatic compounds that were detected by GC–MS (i.e., phenolic compounds, cinnamic acid derivatives, and benzoic acid derivatives), together with the corresponding TPC. Although cumulative percent areas are qualitative, it can be observed that the highest cumulative areas corresponded to EA1–EA3 and M1–M5; these are fractions that were eluted with non-polar eluents. A similar behavior could be observed regarding phenolic compounds, which reached up to 37.5%A, 36.6%A, and 50%A in EA3, M1, and M4, which could be the origin of their high TPC. Opposite to this, M6–M11 presented low cumulative %A for aromatic compounds and particularly for phenolic compounds, which is the origin of the decrease in the as-measured TPC. As can be seen in Table 4, Soxhlet and flash chromatography seemed to be effective in compound fractionation. Thus, benzoic acid derivatives are mainly found in ethyl acetate fractions. Cinnamic acid derivatives are only significant in dichloromethane and ethyl acetate fractions, whereas, in methanol fractions, they could only be detected in M4. This relatively high content in cinnamic acid derivates could be the origin of their relatively high AOA.

The low cumulative areas for aromatics in fractions M6–M11 are in good agreement, with the detection of a significant amount of pentoses and hexoses (Table 3). Saccharides are mainly extracted from the holocellulose fraction of bark in the methanol round, showing that saccharides, at least monosaccharides, can be readily extracted from poplar bark [17,68]. Indeed, the extraction of mannose from barks has already been described using hot water extraction [69,70]. Furan derivatives detected in methanol fractions have also been detected in the hydrolysates of shrub species [71] and as bulk compounds of *Populus tremuloides* bark [25]. 5-hydroxymethylfurfural may also come from the hydrolysis of 5-hydroxymethylfurfural glycosides [26] or from the degradation of hexoses during the drying step. Both furanic compounds, together with furanol, were detected in significant amounts in these fractions, where the presence of saccharides was significant.

Thus, considering both TPC and AOA, together with the isolated masses for each fraction (see SI.3), ethyl acetate and methanol extracts are by far the most promising for the production of bioactive extracts from *Populus salicicaceae* in good mass yields, being 1.1% and 0.6% for EA3 and M4, the highest mass yields with reasonable TPCs and cumulative areas. In the case of methanol, however, the best TPC and cumulative areas were found in M1–M4, whose mass yield was 0.7%, whereas the highest yield corresponded to M5 (1.7%), which, however, presented lower TPCs and cumulative areas (242 μg/mg GAE, 22%A) because of the presence of saccharides.

## 4. Conclusions

The sequential extraction of poplar bark with solvents of increasing polarity produced extracts with good TPC and AAO. The fractionation of these extracts by flash chromatography allowed us to obtain fractions with increased TPC and AOA, reaching up to 608 μg GAE/mg and 793 μg AAE/mg, respectively. A nice correlation between TPC and AOA values was observed. 2,3-dihydrofuran or cyclohexan-1,2-diol from flavonoids and glucosynalates have been identified in most of the fractions. According to qualitative GC–MS, most of the detected aromatic compounds corresponded to phenolic compounds, which may be the origin of the TPC and AOA activity, although cinnamic and benzoic acid derivatives are also present in the dichloromethane and ethyl acetate fractions. Therefore, poplar bark can be considered a promising source of compounds with antioxidant properties using this simple and reliable methodology. Among all those considered, the ethyl acetate extracts seem to be the most promising in terms of extraction yields, TPC, AOA, and the amount of detected phenolic compounds. Future studies must tackle the quantification and isolation of these compounds at larger scales for their potential industrial application.

## Figures and Tables

**Table 1:** Average extraction yields, total phenolic content, and antioxidant activity of the different crude extracts.

Solvent	Average Extraction Yield (wt.%, Dry Basis)	Total Phenolic Content (μg GAE/mg)	Antioxidant Activity (μg AAE/mg)
Hexane	2.1	343	122
Dichloromethane	1.1	172	308
Ethyl acetate	2.8	124	318
Methanol	15.6	84	306
Water	2.6	117	193

**Table 2:** Total phenolic content and antioxidant activity for the different fractions after chromatography of the crude extracts. H, D, EA, and M account for hexane, dichloromethane, and methanol, respectively. Figures account for elution order upon chromatography fractionation, with 1 being the first eluted fraction.

Extract *	Total Phenolic Content (μg GAE/mg)	Antioxidant Activity (μg AAE/mg)
H	343	n.m. **
D	172	308
D2	10	126
D3	149	401
D4	240	630
EA	124	324
EA1	234	499
EA2	371	617
EA3	245	503
EA4	200	468
EA5	12	74
EA6	83	300
EA7	82	337
EA8	45	n.m. **
M	84	261
M1	608	634
M2	2	165
M3	225	713
M4	331	793
M5	242	678
M6	240	568
M7	286	485
M8	102	342
M9	105	358
M10	98	356
M11	67	n.m. **

* Figures correspond to the elution order. ** n.m.: not measured.

**Table 3:** Results of the GC–MS analyses. Relative percentage area for the identified compounds in selected fractions. H, D, EA, and M account for hexane, dichloromethane, and methanol, respectively. Figures account for the elution order upon chromatography fractionation, with 1 being the first eluted fraction.

Retention Time (min)	Peak #	Compound Name	D3	EA1	EA2	EA3	EA4	EA8	M1	M4	M5	M6	M7	M8	M9	M10	M12
		Saccharides	-	-	-	-	-	-	4.3	0.8	1.4	28.2	34.6	37.1	54.6	32.8	-
		Total Aromatic compounds	25.6	37.1	29.6	41.4	14.7	23.1	50.5	51.1	22.3	8.6	9.4	1.1	0.9	4.5	-
		Cinnamic acid derivatives	10.8	1.8	5.1	3.2	1.4	-	-	1.1	-	0.6	0.2	-	-	-	-
		Benzoic acid derivatives	-	1.7	6.6	0.7	-	-	13.9	-	-	-	-	-	-	-	-
		Phenolics	14.8	33.6	17.9	37.5	13.3	23.1	36.6	50	22.3	8	9.2	1.1	0.9	4.5	-
11.96	1	Undecane	-	-	-	-	-	-	-	6.1	8.6	3.8	6.1		9.3	8.3	-
11.66	2	Cyclohexane-1,2-diol	7.7	5.0	6.3	9.1	2.5	-	11.9	14.9	3.7	1.5	1.5	6	0.3	0.8	-
10.22	3	Phenol	-	0.3				-		0.5		-	-	1.1		-	-
13.42	4	Catechol	-	3.2	1.7	2.7	0.9	-		2.6	7.3	-	-	-	-	-	-
13.43	5	Resorcinol	0.8					-	0.2	-	-	-	0.8	-	-	-	-
12.84	6	4H-pyran-4-one-2,3-dihydor-3,5-dihydorxy-6-methyl	-	-	-	-	-	-	-	-	-	-	4.1	2.6	-	2.5	5.0
17.49	7	3-hydroxybenzoic acid		3.6	-	-	-	-	-	8.9	-	-	-	-	-	-	-
14.57	8	Salicylic acid derivatives	3.2	1.4	1.3	2	-	-	0.9		-	-	-	-	-	-	-
20.48	9	Methyl 3-(3-hydroxyphenyl)-2-propenoate	-	-	-	-	-	-	15.2	3.3	-	-	-	-	-	-	-
15.29	10/11/12	4-vinyl-2-ethoxyphenol, 1-(2-hidroxi-5metilfenil)ethanone, 2-hydroxy-6-methylbenzaldehyde	2.0	7.1	2.2	8.6	2.3	2.7	-	12.5	8.8	4.1	4.6	-	-	2.0	-
13.76	13	2,3-dihydrobenzofuran	0.5	4.1	2.8	10	2.5	2.7		12.8		3.1	3.8	-	0.9	2.5	-
17.74	14	Di-tertbutylphenol	1.1	1.6	-	-	4	13.9				-	-	-	-	-	-
17.08	15 E/Z	Iso-eugenol	-	-	-	-	-	-	-	0.5	0.5	-	-	-	-	-	-
16.48	16	Vanillin	1.1	2.1	1.5	-	0.6	-	8	-	0.2	-	-	-	-	-	-
17.62	17	1-(4-hydroxy-3-methoxyphenyl)ethanone	-	-	-	-	-	-	0.7	-	-	-	-	-	-	-	-
18.78	18	Butirovainillone	1.3	3.6	2.3	7.4	2.0	-	-	6.2	5.0	-	-	-	-	-	-
19.53	19/20	4-(4-hidroxy-3-methoxyphenil)-2-butanone/4-(4-hydroxy-3-methoxyphenyl)-2-butanone	1.4	-	0.8	-	-	-	-	-	-	-	-	-	-	-	-
19.57	21	Methyl 4-(4-hydroxy-3-methoxyphenyl)-2-butanoate	-	4.6	-	-	-	-	-	-	-	-	-	-	-	-	-
18.33	22	Vanillic acid	1.8	0.2	0.6	1.4	-	-	-	-	-	-	-	-	-	-	-
16.29	23	3-hydroxy-4-methoxybenzoic acid	-	0.1	-	-	-	-	-	-	0.5						
17.91	24	Methyl-4-hydroxy-3-methoxybenzoate	-	0.1	-	-	-	-	1.5	-	-	-	-	-	-	-	-
16.62	25	Cinnamic acid	10.8	1.8	6.6	5.3	1.4	-	-	1.1	-	0.6	0.2				
16.27	26	Methyl cinnamate	-	-	-	-	-	-	-	-	-	-	-	-	-	-	-
20.83	27	*p*-coumaric acid	-	-	1.5	2.1	-	-	-	-	-	-	-	-	-	-	-
20.51	28	4-(3-hydroxyprop-1-en-2-yl)-2-methoxyphenol	-	-	-	-	1.0	-	-	-	-	-	-	-	-	-	-
20.49	29	Conyferyl aldehyde	-	1.6	1.5	1.2	-	-	-	-	-	-	-	-	-	-	-
19.45/	30	Methyl 3-hydroxycinnamate	-	-	-	-	-	2.9	6.0	-	-	-	-	-	-	-	-
20.20	31m	Methyl 3-(4-hydroxy-3-methoxyphenyl)prop-2-enoate	-	-	0.2	-	-	0.9	1.8	-		0.1	-	-	-	-	-
21.57	31p	Methyl 3-(4-hydroxy-3-methoxyphenyl)prop-2-enoate	0.6	-	-	-	-	-	2.3	-	-	0.7	-	-	-	-	-
16.77	32	2-methoxy-1,4-benzenodiol	-	-	-	-	-	-	-	2.2	-	-	-	-	-	-	-
17.59	33	Methoxybenzoic acid	-	0.6	5.9	-	-	-	0.9		-	-	-	-	-	-	-
19.53	34	Methyl n-methoxybenzoate	-	1.1	0.7	0.7	-	-	13.0		-	-	-	-	-	-	-
19.66	35	Syringaldehyde	1.0	-	-	-	-	-	-	0.5	-	-	-	-	-	-	-

**Table 4:** TPC, AOA, and cumulative percent areas for Phenolic compounds, cinnamic acid, benzoic acid derivatives, and aromatic compounds.

	TPC (μg GAE/mg)	AOA (μg GAE/mg)	Phenolic Compounds (%A)	Cinnamic Acid Derivatives (%A)	Benzoic Acid Derivatives (%A)	Aromatic Compounds (%A)
D3	149	401	14.8	10.8	-	25.6
EA1	234	499	33.6	1.8	0.6	37.1
EA2	371	617	17.9	5.1	5.9	29.6
EA3	245	503	37.5	3.2	0.7	41.4
EA4	200	468	13.3	1.4	-	14.7
M1	608	634	36.6	-	0.9	50.5
M4	331	793	50.0	1.1	-	51.1
M5	242	678	22.3	-	-	22.3
M6	240	568	8.0	0.6	-	8.6
M7	286	485	9.2	0.2	-	9.4
M8	102	342	1.1	-	-	1.1
M9	105	358	0.9	-	-	0.9
M10	98	356	4.5	-	-	4.5
M11	67	n.m. *	-	-	-	-

* not measured.

## Data Availability

Not applicable.

## Supplementary Materials

The following supporting information can be downloaded at: https://www.mdpi.com/article/10.3390/biom12040539/s1, SI.1. Extraction methodology, SI.2. Antioxidant activity, SI.3. Fractionation of extracts. Figure S1. Antiradical activity in blank solutions; Table S1. Physical properties of the selected solvents; Table S2. Fractionation of H extracts; Table S3. Fractionation of D extracts; Table S4. Fractionation of EA extracts; Table S5. Fractionation of M extracts; Table S6. Fractionation of W extracts.
