# Supplementary material for: Extraction of Phenolic Compounds from Populus Salicaceae Bark
Source: Biomolecules. 2022 Apr 2;12(4):539. doi: 10.3390/biom12040539 (PMC9025220; doi:10.3390/biom12040539)
Supplement: Supplementary file 1 [file biomolecules-12-00539-s001.zip › biomolecules-1638689-supplementary.pdf]

Supplementary Material
Extraction of chemical compounds from poplar bark

Elsa Autor, Alfonso Cornejo*, Fernando Bimbela*, Maitane Maisterra, Luis M. Gandía and Víctor Martínez-Merino
Institute for Advanced Materials and Mathematics (INAMAT^2) and Department of Sciences,
Universidad Pública de Navarra (UPNA), Campus de Arrosadía, 31006 Pamplona, Spain;
* Correspondence: alfonso.cornejo@unavarra.es, fernando.bimbela@unavarra.es

Supporting Information

SI.1. Extraction methodology

Table S1. Physical properties of the selected solvents.

Solvent	Boling point (ºC)	Dipole moment (Db)	Dielectric constant
Hexane	69	0	2.02
Dichloromethane	40	1.60	9.08
Ethyl acetate	78	1.78	6.02
MeOH	65	1.70	33.00
Water	100	1.85	78.20

SI.2. Antioxidant activity

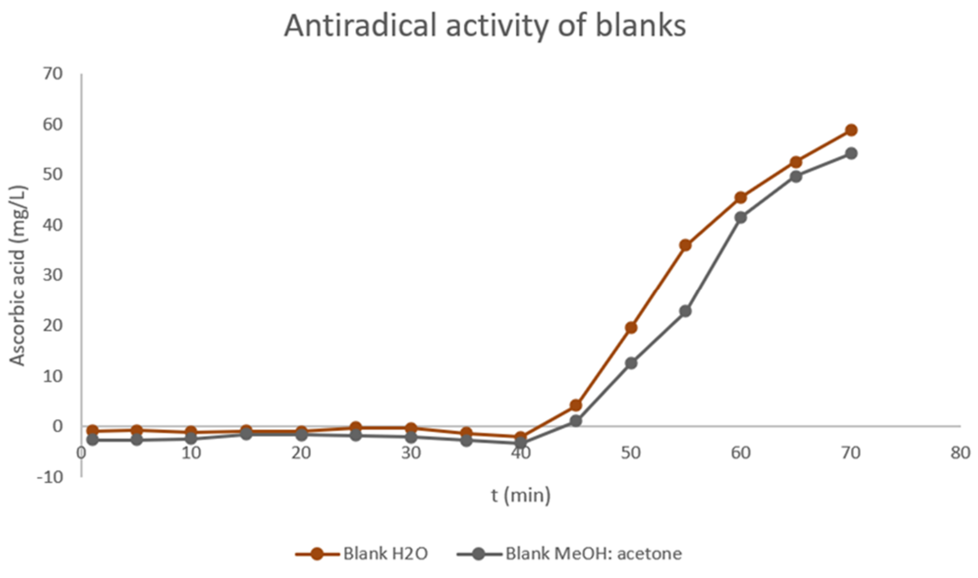

Figure S1. Antiradical activity in blank solutions

SI.3 Fractionation of extracts

Table S2. Fractionation of H extracts

Fraction	#fractions	Total volume/ mL	Eluent
H1	13	95	Hexanes : Ethyl acetate 1:1
H2	3	22	Ethyl acetate
H3	5	37	Ethyl acetate
H4	5	37	Ethyl acetate
H5	4	29	Ethyl acetate
H6	4	29	Ethyl acetate

Table S3. Fractionation of D extracts

Fraction	#fractions	Total volume/ mL	Eluent	Mass/ mg
D1	14	100	DCM: Ethyl acetate 7:3	9.3
D2	2	14	DCM: Ethyl acetate 7:3	19.5
D3	11	76	DCM: Ethyl acetate 1:1	55.3
D4	4	28	DCM: Ethyl acetate 1:1	17.2
D5	4	28	DCM: Ethyl acetate 1:1	49.3
D6	16	112	DCM: Ethyl acetate 1:5	11.2
D7	6	42	Ethyl acetate	48.1
			**Total**	**209**

Table S4. Fractionation of EA extracts

Fraction	#fractions	Total volume/ mL	Eluent	Mass/ mg
EA1	5	62	DCM/MeOH 7:3	192
EA2	4	50	DCM/MeOH 1:3	1057
EA3	4	50	DCM/MeOH 1:3	551
EA4	4	50	DCM/MeOH 1:3	132
EA5	6	75	DCM/MeOH 1:3	54
EA6	4	50	DCM/MeOH 1:3	8
EA7	3	38	DCM/MeOH 1:3	3
EA8	4	50	DCM/MeOH 3;7; MeOH	0
			**Total**	**1997**

Table S5. Fractionation of M extracts

Fraction	#fractions	Total volume/ mL	Eluent	Mass/ mg
M1	16	132	DCM/ AcOEt	24
M2	4	33	DCM/EA 3:7 DCM/MeOH	90
M3	2	16	DCM/MeOH 7:3	7
M4	2	16	DCM/MeOH 7:3	136
M5	4	33	DCM/MeOH 7:3	1749
M6	8	66	DCM/MeOH 7:3	3544
M7	5	41	DCM/MeOH 3:7	1346
M8	4	33	DCM/MeOH 3:7	259
M9	3	25	DCM/MeOH 3:7	84
M10	4	33	DCM/MeOH 3:7	149
M11	1	8	DCM/MeOH 3:7	26
M12	18	214	MeOH and iPrOH	20
			**Total**	**7434**

Table S6. Fractionation of W extracts

Fraction	#fractions	Total volume/ mL	Eluent	Mass/ mg
W1	10	98	AcOEt	54.2
W2	4	39	AcOEt	38.6
W3	2	20	MeOH	35.7
W4	5	49	MeOH	70.2
W5	1	10	MeOH	8.6
W6	4	39	MeOH	31.3
W7	1	10	iPrOH	4.6
W8	4	39	iPrOH	5.1
			**Total**	**248**
